# Simple, Safe, and Cost-Effective Technique for Resected Stomach Extraction in Laparoscopic Sleeve Gastrectomy

**DOI:** 10.1155/2016/7090128

**Published:** 2016-05-05

**Authors:** Serhan Derici, Koray Atila, Seymen Bora, Serkan Yener

**Affiliations:** ^1^General Surgery Department, School of Medicine, Dokuz Eylul University, Balcova, 35340 Izmir, Turkey; ^2^Endocrinology Department, School of Medicine, Dokuz Eylul University, Balcova, 35340 Izmir, Turkey

## Abstract

*Background*. Laparoscopic sleeve gastrectomy (LSG) has become a popular operation during the recent years. This procedure requires resection of 80–90% of the stomach. Extraction of gastric specimen is known to be a challenging and costly stage of the operation. In this paper, we report results of a simple and cost-effective specimen extraction technique which was applied to 137 consecutive LSG patients.* Methods*. Between October 2013 and October 2015, 137 laparoscopic sleeve gastrectomy surgeries were performed at Dokuz Eylul University General Surgery Department, Upper Gastrointestinal Surgery Unit. All specimens were extracted through a 15 mm trocar site without using any special device.* Results*. We noticed one superficial incisional surgical site infection and treated this patient with oral antibiotics. No cases of trocar site hernia were observed.* Conclusion*. Different techniques have been described for specimen extraction. This simple technique allows extraction of specimen safely in a short time and does not require any special device.

## 1. Introduction

Laparoscopic Sleeve Gastrectomy (LSG) has become an increasingly popular bariatric procedure worldwide [1, 2]. This procedure is described as resection of 80–90% of the stomach, leaving only a sleeve of stomach along the lesser curvature. A minimum of 1,100 mL of gastric volume is suggested to be removed to achieve a long term weight loss [3]. This means that there will be a large specimen to be extracted when the resection is completed.

The extraction of the specimen may become a long and challenging stage of laparoscopic surgery. Especially in obese patients, the proportion of the opening for the specimen extraction and the size of the specimen as well as the thickness of the fat tissue between the skin and the fascia are factors that render the extraction more difficult [4].

Different techniques for the extraction of the resected specimen have been described. Some authors suggest using an endobag through a 15 mm trocar or placement of a wound protector [5]. Specimen morcellation or intraabdominal specimen partitioning has also been reported [6]. In 2010, Casella et al. reported a novel technique involving a simple and cost-effective extraction which requires the enlargement of the port site [7].

In the present study, we investigated the effects of a specimen extraction technique we applied without using any wound protector, retrieval bag, or fascial enlargement on wound infection during the early postoperative period.

## 2. Material and Method

All the laparoscopic sleeve gastrectomy procedures performed at the Dokuz Eylul University General Surgery Department, Upper Gastrointestinal Surgery Unit, Izmir, Turkey, between October 2013 and October 2015 were evaluated.

### 2.1. Technique

All procedures were performed under general anesthesia in the modified Lloyd Davies position (thighs parallel to the ground with a 30-degree reverse Trendelenburg position). A single dose of 2 g cefazolin was used for the surgical prophylaxis. Following the placement of one 15 mm, one 10 mm, and three 5 mm trocars, the left lobe of the liver was retracted using a Nathanson liver retractor. The greater curvature was skeletonized using a Ligasure*™* vessel sealing device (Covidien, Norwalk, CT, USA). The skeletonization was started at 4–6 cm to the pylorus and continued until the left crus is reached. Subsequently, a transoral 36 french bougie was placed along the lesser curvature sleeve created using linear staplers EndoGIA® (Medtronic Norwalk, CT, USA). Two sequential 4.8/60 mm green loads were fired for the antrum, followed by 2–4 sequential 3.5/60 mm blue reloads for the remaining corpus and fundus, or Tri-Staple*™* (Autosuture Norwalk, CT, USA) was applied using purple and tan cartridges. The staple line was reinforced with running suture by the V-Loc*™* 180 absorbable wound closure device (Medtronic Norwalk, CT, USA).

**Figure 1:**
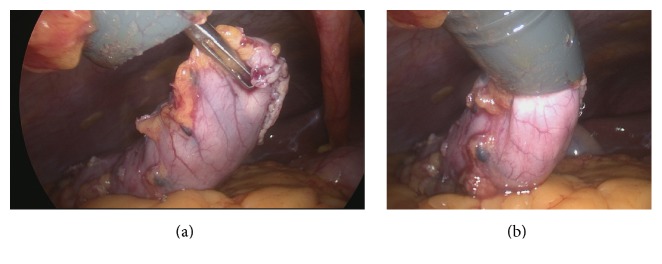
Intraoperative view of specimen grasping (a) and pulling into the 15 mm trocar for extracting (b).

Without any enlargement of the muscle wall at the 15 mm trocar site, the resected stomach was grasped at the caudal tip by a laparoscopic grasper trough the 15 mm trocar. The first 2-3 cm proportion of the resected stomach was pulled into the 15 mm trocar and then the grasper was extracted together with the trocar and the tip of the specimen (Figures 1(a) and 1(b)).

**Figure 2:**
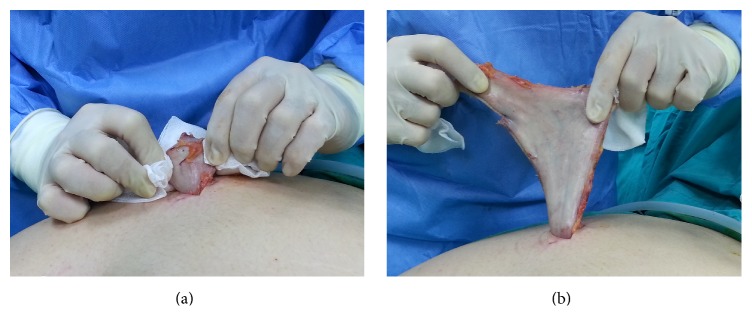
Extraction of the entire specimen via the 15 mm trocar site by gauze sponge.

Using a gauze sponge (to prevent a wall defect) and pulling up alternately the greater curvature and the staple line (twice the greater curvature, once the staple line, repeatedly), the stomach was removed entirely through the 15 mm trocar site defect taking care not to open the staple line (Figures 2(a) and 2(b)). After the extraction of the specimen, the right upper quadrant was routinely irrigated with warm 0.9% saline solution. The silicone suction drain was placed along the sleeve and fascial defect was closed after the extraction was completed. The 10 mm and 15 mm trocar sites were closed with number 0 polydioxanone sutures using the 10 mm and 15 mm trocar pilot guides and suture passer under laparoscopic direct visualization. Two infusion catheters were inserted into the proximal and distal parts of the specimen for filling with saline and to measure the pressure. Specimen was filled with saline until an intragastric pressure of 12 mmHg was reached. The volume of the resected stomach was assessed at 12 mmHg intragastric pressure. All specimens are submitted to pathology for examination.

A prospectively recorded database of 137 patients who underwent LSG procedures was reviewed. The parameters evaluated in this paper included age, sex, body mass index (BMI), diabetes, cardiac and pulmonary disease, resected gastric volume, resected gastric weight, length of the greater curvature, staple line of the specimen, any staple line dehiscence during specimen extraction, types of the staple cartridges, port site infection or wound dehiscence, and incidence of port site hernia.

## 3. Results

The mean age of the patients was 39.49 (±10.02) years and 78.1% were female. The mean preoperative body mass index was 45.9 (±5.96) kg/m^2^ (range: 36–67.2) and 60 patients had at least one comorbidity (DM, pulmonary or cardiovascular disease). Tri-Staples*™* were used for 59 (43.1%) patients; EndoGIA blue and green cartridges were used for 66 (48.2%) patients. For 12 (8.7%) patients, Tri-Staples and EndoGIA blue and green cartridges were used together. The mean resected gastric volume was 1107.1 (±281.29) mL and the mean weight of the specimen was 131.93 (±110) g for the last 98 patients. The other measurements of the specimens are shown in Table 1. We observed two malignant results (gastrointestinal stromal tumor and neuroendocrine carcinoma) at the pathological examination of specimens.

There were three minimal incidents of stapler line dehiscence (1–3.5 cm) during the extraction of the specimen. Nevertheless, none of the patients had trocar site infections. We observed a hematoma at the 10 mm trocar site in one patient (0.72%). There were no cases of trocar site hernia among the 137 patients for a mean follow-up period of 12 months. Only one patient with a 45.5 BMI and DM had an infection at the 15 mm trocar site (0.72%). The infection was treated using empiric antibiotic therapy without any invasive procedure.

## 4. Discussion

The term “surgery-associated wound infections” were modified as surgical site infections (SSI) in 1992 and classified as superficial incisional, deep incisional, and organ/space surgical site infections [8]. Obesity has been observed as the main risk factor for the development of SSIs [9]. The incidence of SSIs after bariatric surgical procedures performed for the treatment of obesity was reported as 10% for open surgery and 3–5% for laparoscopic procedures [10]. At the final stage of the laparoscopic sleeve gastrectomy, which has been the most popular bariatric procedure performed during the recent years, the specimen has to be extracted without raising the incidence of intraabdominal or incisional infection. Wound protectors or retrieval bags are frequently used for this purpose. In order to facilitate the extraction of a specimen of this size using retrieval bags, Mahmood and Silbergleit suggested to morcellate the specimen [6]. In the literature, the rate of incidental malignancies observed during pathological assessment of the specimen after LSG was reported as 0.2% [11, 12]. The method suggested by Mahmood and Silbergleit may complicate pathological examination. In our cohort, we observed one case of Grade 2 neuroendocrine carcinoma (0.7%) and another one with a 5 mm gastrointestinal stromal tumor (0.7%) during the pathological examination of the specimen. The preoperative gastroscopic evaluations of these patients were normal. Based on these results, we do not suggest morcellation.

Specimen extraction techniques without using retrieval bags or wound protectors have also been described to reduce the operating costs. Gorecki et al. described intraabdominal gastric specimen partitioning and extraction of the resected stomach slices through a 15 mm trocar [13]. This technique changes the procedure from a clean-contaminated operation into a contaminated operation. The incidence of surgical site infections is higher for contaminated operations compared to clean-contaminated operations (2.1% versus 3.3%) [14]. Moreover, intraabdominal infection development has been described as an independent risk factor for development of incisional surgical site infections [15]. Based on these data, we do not suggest this technique.

Casella et al. reported a new extraction technique that does not require any special devices [7]. In this technique, the fascia is enlarged by finger dilatation at the 15 mm trocar site. The caudal tip of the resected stomach is grasped and pulled into the 15 mm trocar. The specimen is removed entirely through the enlarged 15 mm trocar site with the help of the Kocher clamps. They reported the incidence of wound infection as 1.2%. Our experience confirms the results reported by this author.

In our technique, we avoided trocar site enlargement, since the tissue trauma increases postoperative pain and the incidence of trocar site hernia [16]. Furthermore, we did not use Kocher clamps to avoid specimen laceration or microperforation. We observed only one case of wound infection (0.72%) through our technique. The patient with wound infection was 61 years old and she had DM and hypertension with a 45.5 kg/m^2^ BMI. The infection was treated using empiric antibiotic therapy without any invasive procedure. We did not observe any trocar site hernias among the 137 patients for a mean follow-up period of 12 months.

In our method, we use a 15 mm trocar with a sharp blade and place this trocar at the lateral aspect of the rectus and maintain a full muscle relaxation before and during the extraction. We thus can extract the specimen using a gauze sponge without the need for a fascial enlargement (if a 12 mm trocar is used facial enlargement may be required). Reducing the trauma at the trocar site by avoiding any fascial enlargement and refraining from the use of the Kocher clamp, we avoid any microperforations that may occur in the specimen. The extraction is always followed by an irrigation of the intraabdominal right upper quadrant using saline. We believe that the low incidence of wound infection in our series is a result of these modifications (0.72%).

Extractions using wound protectors or specimen bags have no significant superiority to the technique we performed in terms of infection or hernia development [4, 17, 18]. Through the extraction performed without using a wound protector or specimen bag, 180€ is saved per patient without an increase in the ratio of the surgical site infections [7]. We are of the opinion that reduced costs may be of significance for the facilities where a large number of operations are performed.

## 5. Conclusion

This simple and cost-effective technique that does not require any special devices and has a very low incidence of wound infection can be safely applied.

## Figures and Tables

**Table 1:** Patient characteristics and specimen measurements.

Age (mean)	39.49	(±10.02)

Sex		
Male	30	(21.9%)
Female	107	(78.1%)

Body mass index (mean)	45.9	(±5.96)

Dm		
+	38	(27.7%)
−	99	(72.3%)
Pulmonary comorbidity		
+	15	(10.9%)
−	122	(89.1%)
Cardiovascular comorbidity		
+	36	(26.3%)
−	101	(73.7%)

Specimen volume (mL)	1107.1	(±281.29)

Specimen weight (g)	131.93	(±110)

Length of staple line (cm)	27.18	(±3.48)

Length of greater curvature (cm)	51.27	(±6.30)
